# Rapid Discrimination and Authentication of Korean Farmstead Mozzarella Cheese through MALDI-TOF and Multivariate Statistical Analysis

**DOI:** 10.3390/metabo11060333

**Published:** 2021-05-21

**Authors:** Sujatha Kandasamy, Jayeon Yoo, Jeonghee Yun, Han-Byul Kang, Kuk-Hwan Seol, Jun-Sang Ham

**Affiliations:** Animal Products Research and Development Division, National Institute of Animal Science, Rural Development Administration, Wanju-gun 55365, Korea; sujirda2019@korea.kr (S.K.); yjy1172@korea.kr (J.Y.); rsvped@korea.kr (J.Y.); khb1771@hanmail.net (H.-B.K.); seolkh@korea.kr (K.-H.S.)

**Keywords:** domestic cheeses, mass spectrometry, geographical origin, protein fingerprints, cheese authentication

## Abstract

Geographical origin and authenticity are the two crucial factors that propel overall cheese perception in terms of quality and price; therefore, they are of great importance to consumers and commercial cheese producers. Herein, we demonstrate a rapid, accurate method for discrimination of domestic and import mozzarella cheeses in the Republic of Korea by matrix-assisted laser desorption/ionization time-of-flight mass spectrometry (MALDI-TOF MS). The protein profiles’ data aided by multivariate statistical analysis successfully differentiated farmstead and import mozzarella cheeses according to their geographical location of origin. A similar investigation within domestic samples (farmsteads/companies) also showed clear discrimination regarding the producer. Using the biomarker discovery tool, we identified seven distinct proteins, of which two (*m*/*z* 7407.8 and 11,416.6) were specific in farmstead cheeses, acting as potential markers to ensure authentication and traceability. The outcome of this study can be a good resource in building a database for Korean domestic cheeses. This study also emphasizes the combined utility of MALDI-TOF MS and multivariate analysis in preventing fraudulent practices, thereby ensuring market protection for Korean farmstead cheeses.

## 1. Introduction

Food authentication procedures are gaining much importance in the international food market because of increasing consumer awareness regarding food quality and safety. Market globalization and industrialization of food products forced the industry to become very competitive and financially profitable by creating more opportunities in adulteration/false labeling [1,2]. The growing demand for high-quality foods, the desire for new delicate products or foods having specific organoleptic traits, and the consumer’s ability to pay a high price provoke more vulnerability in counterfeiting [3]. The increased exposure of food scandals and their adverse effects on public health in recent years has urged consumers to demand more evidence on the authenticity and quality of popularized foods. Substitution of rare/expensive ingredients with cheaper ones to attain high financial gains is crucial in animal-origin foods, especially in dairy products [3,4].

Cheese is the most prominent dairy food that has become the subject of counterfeiting in the global market. The premium price for specialty cheeses and fluctuation in milk availability make it profitable for the cheese manufacturers to engage in adulteration and reduce production costs [5,6]. Food frauds in cheeses entail several undesirable consequences, and they are consumed widely from infants to aged people in many countries. Fraudulent substitution of low-cost milk and/non-milk fat during the cheese production and mislabeling of cheese composition/geographical origin can expose consumers to potential allergens and deceive consumers’ gratification [1,7,8]. The cheese producers must compulsorily provide the species origin of milk as few consumers evade cow’s milk for allergic/intolerance disorders and ethical/religious reasons [5,9]. Because of these legal aspects and consumerism and reliability in the dairy industry, authentication becomes mandatory to define the uniqueness against frauds or commercial disputes [2,10].

Cheese consumption in the Republic of Korea (ROK) is steadily increasing over the decades, outpacing domestic production. To meet the growing demand in the consumer market and food sectors, the country highly depends on imported cheeses from the United States, the European Union, New Zealand, and Australia [11]. Despite the high market price, growing health concerns towards food safety and a healthy lifestyle have forced many Koreans to rely upon domestic farmstead cheeses, consequently increasing the proportion of domestic dairy farms [12,13]. The high production costs, demand, and uniqueness drive the necessity to authenticate farm products, thereby protecting consumers against the deception of adulterant and imitant cheeses. To identify mislabeling about the geographical origin for cheeses is an international issue gaining increased attention, as the specialty cheeses or cheeses produced in particular regions relate to a high price for their superior and precise attributes [1,8]. Most of the authentication techniques in dairy products depend upon the analysis of fatty acid profiles or milk protein content. In last few years, matrix-assisted laser desorption/ionization time-of-flight (MALDI-TOF) mass spectrometry, a powerful analytical tool, is gaining increased attention to detect adulteration in milk and cheeses [6,7,14,15]. This tool is already well-known for microbial identification [16] and biomarker discovery in cancer diagnosis [17] owing to its high precision, rapidity, simple operation, and great sensitivity, even in low molecular weight samples [15]. More recently, protein/peptide fingerprints from MALDI-TOF is demonstrated for effectively discriminating the geographical origin or species in foods, including kimchi [18], wine [19], honey [20], mushrooms [21], vegetable oil [22], and meat and gelatin [23].

In this study, our goal was to employ MALDI-TOF MS to determine the protein fingerprints of domestic and import mozzarella cheeses, as well as to combine multivariate statistical analysis to discriminate the samples on the basis of geographical origin. To the best of our knowledge, we are the first to analyze the protein profiles in the domestic mozzarella cheeses of the ROK. Moreover, identifying potential markers allows for functional application in safeguarding the authenticity and traceability of the farmstead cheeses.

## 2. Results and Discussion

### 2.1. Spectra of Mozzarella Cheese Samples

**Figure 1:**
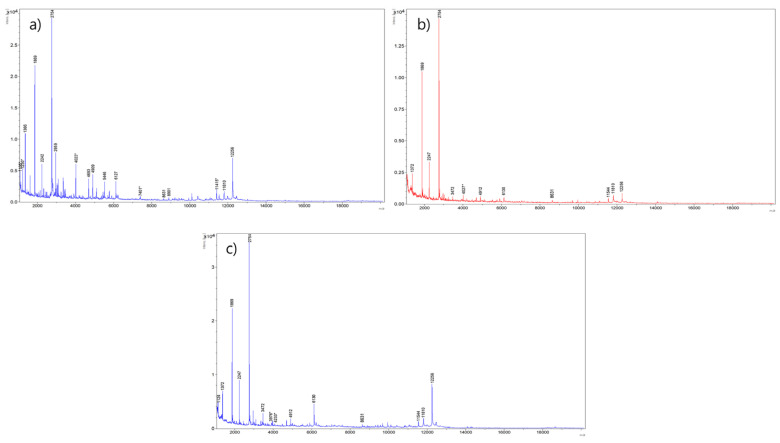
Representative MALDI-TOF mass spectra in the range from 1100 to 18,000 *m*/*z* for mozzarella cheeses from Korean farmstead (**a**), Korean company (**b**), and imports (**c**) in the Republic of Korea. MH^+^ signals corresponding to specific peaks identified in this study are marked with an asterisk.

Cheese is growing as a part of the Korean dietary culture, and there is a growing demand for the mozzarella type for its wide application in foodservice and food processing sectors. MALDI-TOF has been suggested as a valuable tool in the investigation of milk and cheese protein profiles. Few studies have shown MALDI-TOF suitability to evaluate fraudulence in commercially produced water buffalo mozzarella cheese [24] and Pecorino cheese [5]. To the best of our knowledge, there is no research on protein profiles of the domestic cheeses produced in the ROK. Herein, we attempted to develop a direct method using MALDI-TOF MS to characterize the protein profile and to discriminate the mozzarella cheeses (from cow milk) according to geographical origin. For this purpose, cheese samples from 24 domestic farms, 9 domestic companies, and 10 import types were analyzed for their protein profiles, and a representative example from each type is illustrated in Figure 1. The *y*-axis corresponds to the ion intensity (arbitrary units), while the mass/charge ratio (*m*/*z*) is represented on the *x*-axis. Peaks with *m*/*z* from 1000 to 5000 can be well observed.

The mass spectra and protein profiles clearly define the complexity of proteolysis; further, innate enzymes in cheese and thermal events hydrolyze the casein fractions into several diverse peptides [7,25]. Moreover, the pH gradient developed during the cheese ripening process imparts favorable or unfavorable conditions for the activity of the enzymes involved in the ripening process that corroborates with the complexity of proteolytic process [25]. The *m*/*z* peaks at 1869.6, 2754.8, 3470.6, 4478.6, 4693.1, 4909.1, 6127.5, 7049.6, 8631.3, 11,810.9, and 12,254.5 were common in all the mozzarella samples used in this study. The results are also in good agreement with the earlier findings of Rau et al. [26] and support the presence of these signals only in mozzarella cheeses produced from cow’s milk.

The proteins from MALDI-TOF spectra can be determined and quantified by identifying the several peaks and assigning them to specific proteins on the basis of the protein molecular mass data published earlier. However, individual proteins can deviate from the exact position of the peak, and subsequently their molecular mass due to genetic and environmental factors, in particular, the processing conditions of milk (thermal denaturation and proteolysis) can modify individual protein structure [14]. Accordingly, the *m*/*z* peaks 1869.6 and 2754.8 showing the higher relative intensities in all the samples can be assigned to a_s1_-CNf(1–16) and a_s1_-CNf(1–23) fragments corresponding to *m*/*z* 1877 and 2764, respectively, mentioned in previous studies [27,28,29,30]. The a_s1_-CNf(1–23) recognized for immunomodulatory and antimicrobial potential is found mainly in casein-hydrolyzing peptidases from the starter microorganisms [25,31].

Statistical analysis of all the peak intensities between the groups (farm/company/import) using one-way ANOVA revealed a total of 17 significant peaks (ranging from *m*/*z* 1231.80 to 14,092.60) (Table 1). For simplicity, only peaks of statistical significance are discussed. The representative average intensities and standard deviation for the above-mentioned peaks in each sample group are shown in Table 1. The variation can be attributed to the fact that the cheese samples used in this study were obtained from different producers and different geographical locations, as well as being produced at different intervals. The protein profiles and their intensities in the cheeses are greatly influenced by the different production protocols of each production unit [2,9]. For multiple comparisons between the groups, we submitted *p*-values obtained from the ANOVA to Tukey’s post hoc analysis in GraphPad Prism 6.0 software. Pairwise comparisons between the groups revealed higher significance for farmstead samples against both domestic companies and imports. Four peaks in farmstead samples (*m*/*z* 1231.80, 4025.02, 4909.33, and 10,426.62) were significantly different (**** *p* < 0.0001) in comparison to samples from imports and domestic companies. On the other hand, the significance was found to be much lower in the pairwise comparison between company and import groups.

### 2.2. Discrimination of Cheese Samples from Farm and Import

The statistical analysis of data representing the peptide profile is a powerful approach that can be applied to verify the origin of cheese samples. With the aim to obtain precise information about the peptides responsible for the discrimination of groups (farmstead/company/import) according to producer/geographical origin, we used a combination of univariate (volcano plot) and multivariate analyses (PLS-DA).

The volcano plot analysis between the cheese samples from farmstead and import (*p* < 0.01, fold ≥ 1.5) revealed 21 peaks as statistically significant, of which 12 were downregulated and 9 were upregulated (Table 2).

**Figure 2:**
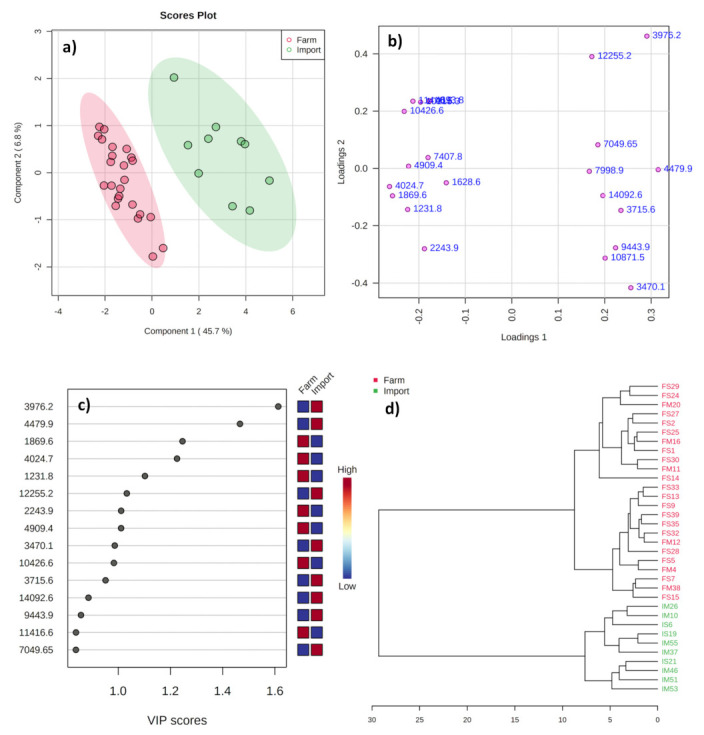
Score (**a**), loading (**b**), and VIP (**c**) plots from the PLS-DA and HCA (**d**) analyses for the mass spectra of mozzarella cheeses from domestic farmsteads and imports commercially available in the Republic of Korea.

The discriminant analysis by PLS-DA (Figure 2) applied between the same groups explained a total variance of 52.5%. The score plot (Figure 2a) shows a clear differentiation between farmstead and import samples, indicating higher variation in their mass data and protein profiles/intensities. Each point in the score plot represents an individual sample. The farmstead samples were found to be tightly clustered inside the Hotelling T2 ellipse (95% confidence region), indicating they share major similar profiles. In contrast, the import samples are sparsely located from each other inside the ellipse. This can be attributed to the fact that the import samples used in this study were obtained from different geographical locations and produced at different time intervals using different procedures. The corresponding loading plot (Figure 2b) presents variables that define the samples in score plot, whereas important variables for group discrimination were certainly in higher positive and negative loadings of PC1. The samples located on the right side of the score plot tended to have higher relative intensities of proteins on positive loadings and a lower abundance of proteins on negative loadings. Interestingly, the variables identified as discriminative were the same as those identified by univariate analysis as having a trend toward being different between the two groups. The variation in climatic conditions, feed type, grassland, and phenological stages influences the protein profiles of the milk, which in turn influences the protein composition of the cheeses. These results show that the geographical origin (and indirectly the environment and diet) plays a key role in the peptide profile of cheeses [2].

The VIP plot from the PLS-DA enables selection of the most essential variables in discrimination on the basis of their VIP scores (VIP >1) [32,33]. Ten peak lists (Figure 2c) were selected as potential classifiers, among which intensity of six peaks (*m*/*z* 1869.6, 4024.7, 1231.8, 2243.9, 4909.4, and 10,426.6) was higher in farmstead samples, while four (*m*/*z* 3976.2, 4479.9, 3470.1, and 12,255.2) were higher in imports. In general, every classification analysis including PLS-DA needs strict validation as it can easily overfit the data. Therefore, the cross-validation method is crucial to measure the predictive accuracy and reliability of the model with limited samples. In our study, the performance of the PLS-DA model was validated through 10-fold cross-validation (10-fold CV). The cross-validation method was validated using the two parameters (R^2^X and Q^2^), where R^2^X and R^2^Y signify the “goodness of fit” for “predictors” and “responses”, respectively, and Q^2^ indicates the “goodness of prediction”. The model is said to have a good predictability when both the parameters are approaching 1 [32,33]. The R^2^X and Q^2^ of the PLS-DA model were 0.919 and 0.817, respectively, representing a good predictive ability.

Finally, hierarchical clustering analysis (HCA) was performed in order to monitor the related clusters and sub-clusters visualized in a dendrogram graph, in which the samples with maximum similarities were clustered preferentially [32]. The dendrogram shown in Figure 2d was obtained by scaling the same variables used for PLS-DA and applying a complete linkage to Euclidean distance between the two groups. The results confirmed the clear separation of cheese samples (farm and import) into two overall clusters, visually representing the variation in geographical origin. The overall results confirm the practical possibility of using protein profiles as a screening tool in discrimination of the cheeses according to geographical origin.

### 2.3. Discrimination of Cheese Samples from Company and Farmstead

The volcano plot analysis (*p* < 0.01, fold ≥ 1.5) between the cheese samples produced in the Korean companies and farmsteads revealed 18 significant peaks, among which 8 were downregulated and 10 were upregulated (Table 2).

**Figure 3:**
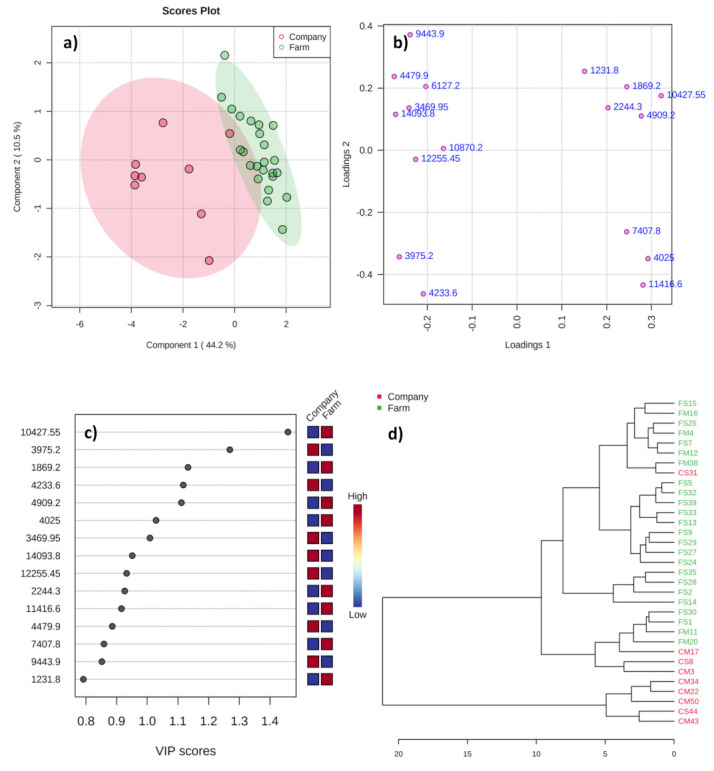
Score (**a**), loading (**b**), and VIP (**c**) plots from the PLS-DA and HCA (**d**) analyses for the mass spectra of mozzarella cheeses from domestic companies and farmsteads commercially available in the Republic of Korea.

The PLS-DA analysis (Figure 3) explained a total variance of 54.7% (Figure 3a) with a slight overlapping between the groups. It can be related to the raw milk composition used for cheese production in both groups being from the same geographical location of origin. One farmstead sample was found to be lying outside the Hotelling T2 ellipse (Figure 3a), while most of the other farmstead samples were tightly clustered together inside the ellipse. In terms of samples from domestic companies, some were sparsely located inside the ellipse, while some clustered together. On the contrary, a complete separation of variables on positive and negative sides of the loading plot (Figure 3b) was evident. The variables located in the loading plot’s negative and positive side represented higher intensities of proteins for samples on the left (domestic companies) and right (farmstead) sides of the score plot.

The variables identified as discriminative in the loading plots were identical to those identified by univariate analysis as having a trend toward being different between the two groups. The variation between the samples from farmsteads and domestic companies can be allied to the usage of imported curds in cheese production by domestic companies. The result agrees with the findings of Feeney et al. [33] that starter culture can adversely affect the composition of small peptides and amino acids in mozzarella cheese. Moreover, several studies have revealed the influence of starter composition on the cheese characteristics and quality [34,35]. The VIP plot displayed seven potential proteins (Figure 3c) as potential classifiers (VIP >1)—three (*m*/*z* 10,427.6, 1869.2, and 4909.2) of them showed higher intensities in farmstead cheeses, and four (*m*/*z* 3975.2, 4233.6, 3470, and 14,093.8) higher in samples from domestic companies. The cross-validation of the PLS-DA model reported R^2^X and Q^2^ as 0.735 and 0.683, respectively. These results expose the influence of starter cultures and raw milk composition on the peptide profile of domestic cheeses.

The dendrogram of HCA analysis (Figure 3d) displays four samples from Korean companies clustered in the same clade of the farmstead samples, while remaining samples from the domestic companies clustered as a separate clade. The variation within the cheese samples from domestic companies can be related to the differences in the cheese processing methodology and starter culture composition [34,35]. The results collectively demonstrate the ability of MALDI-TOF protein profiles in discrimination of the domestic cheeses from farmstead and companies, even though both the producers utilize raw milk from the same geographical location of origin.

### 2.4. Discrimination of Cheese Samples from Company and Import

The volcano plot analysis (*p* < 0.01, fold ≥ 1.5) between cheese samples from domestic companies and imports revealed only three significant signals, of which two were downregulated and one was upregulated (Table 2).

**Figure 4:**
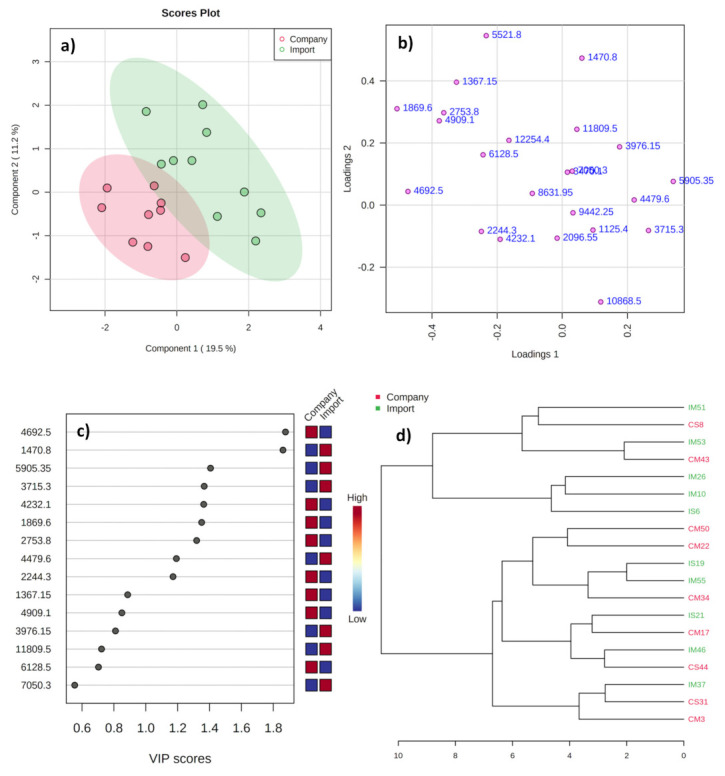
Score (**a**), loading (**b**), and VIP (**c**) plots from the PLS-DA and HCA (**d**) analyses for the mass spectra of mozzarella cheeses from domestic companies and imports commercially available in the Republic of Korea.

The PLS-DA (Figure 4) score plot between the two groups shows partial overlapping, with a total variance of 30.7% (Figure 4a). It is worth mentioning that the total variance decreased < 50% in comparison with earlier plots. Moreover, the variables in loading plots (Figure 4b) did not display clear separation between the groups. These results indicate that the peptide profiles in the cheese samples between the two groups shared major similarities. This may have been due to the usage of imported curds in domestic companies for cheese production. The impact of geographical location of origin of the starter cultures on peptide profiles has been confirmed earlier in a few cheese varieties [34,35]. The VIP plot (Figure 4c) shows nine of the most potential variables in the group discrimination, of which five (*m*/*z* 4692.5, 4232.1, 1869.6, 2753.8, and 2244.3) were higher in samples from domestic companies, while four (*m*/*z* 1470.8, 5905.4, 3715.3, and 4479.6) were higher in imports. The R^2^X and Q^2^ of the PLS-DA model were 0.943 and 0.305, respectively, indicating a weak model.

The result of the HCA analysis visualized as a dendrogram also shows mixed clustering of samples from import with company (Figure 4d). As stated earlier, the mixed clustering might have been due to the influence of import starters employed in domestic companies for cheese production, which contributed to a major similarity of the peptide profiles between the two groups. Previous research works stated that the microbial composition of the traditional water buffalo mozzarella [34] and Caciocavallo Silano [35] cheeses are found to be highly influenced by the natural whey starters used in their production and are closely related to the geographical origin of the starters. Therefore, the peptide profiles of cheeses from the Korean companies did not differ greatly with the import samples, even though the former ones utilize native milk for cheese production. These reasons perhaps explain why the cheese samples from domestic companies and import were clustered in the PLS-DA (loading plot) and HCA graphics. Overall, the statistical analyses showed a low and insignificant discrimination potential between the groups, indicating a major share of similar protein profiles/intensities due to the usage of imported curd as a starter in cheese production by the domestic companies.

### 2.5. Potential Marker Proteins in Discrimination of Cheeses

The application of MALDI-TOF MS in dairy products mainly focuses on the attention towards using intact proteins as a marker for adulterations [6]. Identification of peptides specific to milk species has been unveiled as a convenient approach for detection of fraudulence in milk and cheeses [1,7,8]. This study focused on the ability to use specific proteins as a biomarker fingerprint for authentication and discrimination of the mozzarella cheeses according to the producer/geographical origin, which was determined using the biomarker discovery (inter-class analysis) tool in Mass-Up software. For that, the *m*/*z* peaks with a q-value <0.2 calculated by Benjamini–Hochberg FDR with 100% detection thresholds were selected as potential markers with the most significant discriminatory power as they denote the presence of specific peaks in the sample while not in others.

In this study, a total of seven peaks (*m*/*z* 1101.5, 1231.8, 3976.2, 4024.7, 4233.6, 7407.8, and 11,416.6) were selected as markers on the basis of their discrimination power (Table 3). Five peaks were able to be assigned to protein mass available in earlier studies [27,28,36,37,38,39,40] associated with cheese/dairy products (Table 3). Interestingly, the remaining two unidentified peaks at *m*/*z* 7407.8 and 11,416.6 were specific to Korean farmstead cheeses. The three peaks at *m*/*z* 1101.5, 1231.8, and 4024.7 found in all Korean domestic (farmstead and companies) cheeses were absent in import samples, entailing variation in raw milk composition. Likewise, two peaks at *m*/*z* 3976.2 and 4233.6 common in cheese samples from domestic companies and import samples were not found in farmstead cheeses. These specific peaks can serve as robust markers to enable effective and high-reliable identification of cheeses originating from both Korean farmstead and companies, as well as from other countries. Hence, the method utilized herein can be applied to ascertaining the geographical origin of cheeses samples. The comparison of predicted and theoretical *m*/*z* confirmed that the selected peptides can serve as good candidates in the authentication of the cheeses. Therefore, constructing a database of mass spectra of the cheeses available in the Republic of Korea together with their geographical facts could allow for the identification of geographical location of origin of cheese samples within minutes.

## 3. Materials and Methods

### 3.1. Sample Collection

For this study, we used a total of 43 mozzarella cheese samples (*n* = 43) available commercially in the ROK and assorted into three categories according to the producers, namely, farmsteads (F), companies (C), and imports (I). The farmstead cheeses (*n* = 24) were purchased directly from dairy farms, while samples of domestic companies (*n* = 9) and imports (*n* = 10) were purchased via online and/retail markets. All samples were analyzed within a week or before the end of the shelf-life period.

### 3.2. Sample Preparation

The sample preparation for protein profiling in MALDI-TOF MS was based on the method described by Ham et al. [41]. For dissociation of the caseins and insoluble hydrolysis products, we homogenized each cheese sample (1 g) using 2.5 mL reduction buffer (pH 8.0; 73 mg of trisodium citrate dehydrate + 38 mg of dl-DTT in 37.5 mL of 8 M urea) and left for at least 1 h at room temperature. After centrifugation (15,000× *g*, 2 min), the clear supernatant (2 μL) was mixed with a matrix solution (2 μL) [sinapinic acid dissolved in 30% trifluoroacetic acid acetonitrile mixture (7 mL 0.1% trifluoroacetic acid + 3 mL acetonitrile)] in a 1:1 ratio. Approximately 1 μL of the mixture was hand-spotted onto the ground steel MALDI target plate (Bruker Daltonics, Bremen, Germany). The droplet was allowed to air dry at room temperature, and then the plate was inserted into the mass spectrometer.

### 3.3. Mass Spectrometry

The acquisition of the MALDI-TOF spectra from the cheeses was made using a Microflex TOF mass spectrometer (Bruker Daltonics, Germany) equipped with a pulsed N_2_ laser (337 nm, 3-ns pulse duration). The spectrometer was run with the default parameter settings: positive linear mode, 60 Hz laser frequency, ion source 1 voltage: 20 kV, ion source 2 voltage: 18 kV, and mass range of *m*/*z* 1000 to 20,000 using the FlexControl 3.0 software (Bruker Daltonics, Germany). Three independent spectra were collected manually for each cheese sample to verify the mass accuracy and reproducibility, which was always within the range of 0.5 and 1%. The external mass calibration was measured daily using the Protein Calibration Standard I kit (Bruker Daltonics, Bremen, Germany).

### 3.4. Data Acquisition and Processing

Processing of the mass spectra data files was performed using the Flex Analysis 3.0 software (Bruker Daltonics, Bremen, Germany), and the raw data files were converted to mzmL format (*m*/*z*, intensity lists) using ProteoWizard 3.0 MSConvert [42]. The mzmL files were then imported into the Mass-Up open software (Mass-Up, Vigo, Spain) [43] and subjected to analysis, which included (i) intensity transformation (none), (ii) smoothing (none), (iii) baseline correction (Snip algorithm), and (iv) standardization (total ion current). Peak detection was achieved using MALDIquant (a multifaceted statistical package method) with a signal-to-noise ratio of 3, half-window size of 60, and minimum peak intensity (0.001). Peaks were matched through intra-sample and inter-sample matching (MALDIquant: tolerance (0.002), without selecting the generate consensus spectrum). Additionally, to improve the analysis, we applied quality control to detect and remove low-quality spectra.

### 3.5. Statistical Analysis

The pre-processed mass spectral data containing *m*/*z* values and intensity of the peaks obtained from the Mass-Up was subjected to statistical analysis.

The univariate and multivariate analyses were performed in the online statistical software MetaboAnalyst 5.0 (https://www.metaboanalyst.ca/, accessed on 4 January 2021). Peak lists with a statistically significant difference in terms of signal intensity/mass value were determined through volcano plot (univariate analysis). For visualization and classification of cheese samples according to geographical origin, multivariate analysis using partial least-squares discriminant analysis (PLS-DA) was applied. Data normalization was performed by sum and scaled using the Pareto method. The PLS-DA is a supervised tool that aids in selecting the most relevant variables for sample discrimination, according to the variable importance in the projection score (VIP score > 1). The model was validated by multiple correlation coefficients (R^2^) and cross-validation (Q^2^) parameters.

## 4. Conclusions

This study demonstrated the protein profiles of domestic mozzarella cheeses produced in the ROK for the first time. This rapid and straightforward method was mainly developed to identify food fraud and counterfeiting in Korean farmstead cheeses. Combining MALDI-TOF mass fingerprint data with multivariate statistical analysis discriminated farmstead and import cheeses according to geographical origin. Interestingly, we observed huge differences within the protein profiles of domestic cheeses produced on farms and companies, although both producers were of similar geographical origin. The usage of imported curd as a starter in domestic companies greatly influenced their cheese protein profiles. The outcome of specific proteins in this preliminary study allows for further research on safeguarding Korean domestic cheese authenticity and traceability towards ascribing protected designation of origin. Furthermore, extensive cohort studies are necessary to verify the reliability and reproducibility of protein biomarkers as they may have a significant effect on proteolytic activity, starter materials, and processing conditions. It is eminent that peptides derived from the casein hydrolysis may present multiple biological activities. Hence, identification and characterization of the two unknown proteins designated as biomarkers in the farmstead samples and analyzing their biological activities can make the farmstead cheeses capable of being designated as functional food from a consumer’s health perspective.

## Figures and Tables

**Table 1:** Average intensities and statistical comparison of the peak lists in cheese samples from three different producers (farm/company/import).

*m*/*z*	Average of Peak Intensities (Arbitrary Units)						Anova *p*-Value ^a^	FDR ^a^	Tukey’s HSD Test ^b^		
	Farm (*n* = 24)		Company (*n* = 9)		Import (*n* = 10)				Farm/Import	Farm/Company	Company/Import
	Mean	SD	Mean	SD	Mean	SD					
1231.80	8.24	4.68	2.57	2.81	0.67	0.72	5.2 × 10^−9^	8.9 × 10^−8^	(****)	(****)	ns
1628.63	5.08	3.21	1.70	2.17	2.36	3.40	1.2 × 10^−3^	2.8 × 10^−3^	(*)	(**)	ns
1869.42	50.07	30.80	22.47	31.83	10.55	20.41	2.6 × 10^−5^	1.5 × 10^−4^	(****)	(*)	ns
2244.32	14.36	6.43	7.51	8.19	11.63	27.30	6.9 × 10^−5^	3.3 × 10^−4^	(****)	(*)	ns
3715.46	0.67	1.30	1.22	1.65	5.98	13.34	1.2 × 10^−2^	2.5 × 10^−2^	(**)	ns	ns
3976.18	0.44	0.00	4.86	4.80	4.50	2.52	1.4 × 10^−8^	1.6 × 10^−7^	(***)	(***)	ns
4025.02	7.59	7.30	1.23	2.16	0.35	0.00	2.1 × 10^−7^	1.8 × 10^−6^	(****)	(****)	ns
4232.10	0.30	0.00	1.41	1.25	0.62	0.68	1.7 × 10^−4^	6.5 × 10^−4^	ns	(***)	(*)
4479.91	0.99	1.44	2.51	2.14	3.17	1.74	3.6 × 10^−4^	1.1 × 10^−3^	(***)	ns	ns
4693.16	7.74	5.01	2.76	1.89	1.33	1.54	2.2 × 10^−4^	7.4 × 10^−4^	(***)	ns	ns
4909.33	10.52	5.48	2.75	2.24	1.74	1.33	8.2 × 10^−7^	5.6 × 10^−6^	(****)	(****)	ns
7049.74	0.36	0.37	1.22	1.98	1.36	1.30	2.4 × 10^−2^	4.8 × 10^−2^	(*)	ns	ns
7407.80	1.37	1.54	0.15	0.00	0.15	0.11	1.6 × 10^−4^	6.5 × 10^−4^	(**)	(**)	ns
10,115.31	1.16	1.27	0.29	0.49	0.13	0.00	1.1 × 10^−3^	2.7 × 10^−3^	(**)	(*)	ns
10,426.62	1.82	0.76	0.36	0.51	0.54	0.68	3.5 × 10^−9^	8.9 × 10^−8^	(****)	(****)	ns
11,416.56	2.94	3.70	0.32	0.11	0.32	0.00	6.0 × 10^−4^	1.7 × 10^−3^	(**)	(**)	ns
14,092.60	0.29	0.32	0.78	0.55	0.63	0.45	9.7 × 10^−3^	2.2 × 10^−2^	ns	(*)	ns

^a^—ANOVA *p*-value, FDR obtained using the one-way ANOVA analysis in the software MetaboAnalyst 5.0. ^b^—Tukey’s HSD test was performed with ANOVA *p*-values in GraphPad Prism 8.0 (* *p* < 0.05, ** *p* < 0.01, *** *p* < 0.001, **** *p* < 0.0001; ns = not significant).

**Table 2:** The discriminating *m*/*z* peaks for the mozzarella cheese samples from farmstead, company, and imports in the Republic of Korea.

*m*/*z*	Farmstead/Import		*m*/*z*	Company/Farmstead		*m*/*z*	Company/Import	
	*p*	FC		*p*	FC		*p*	FC
3976.2	7.91 × 10^−12^	−3.22	10,427.6	7.75 × 10^−9^	−2.66	1470.8	0.0128	−1.96
1869.6	2.60 × 10^−8^	2.03	4233.6	3.87 × 10^−6^	2.34	4692.5	0.0411	1.05
1231.8	1.44 × 10^−7^	2.64	3975.2	7.21 × 10^−6^	2.65	5905.4	0.0795	−1.29
4909.4	3.10 × 10^−7^	2.11	1869.2	1.79 × 10^−5^	−1.18			
4479.9	1.17 × 10^−6^	−2.69	12,255.5	4.43 × 10^−5^	1.83			
10,426.6	1.65 × 10^−6^	1.76	4909.2	0.0001	−1.37			
4024.7	2.15 × 10^−6^	3.25	14,093.8	0.0012	1.76			
12,255.2	7.19 × 10^−6^	−1.77	3470.0	0.0027	2.30			
7049.7	0.0013	−2.25	9443.9	0.0041	1.47			
3715.6	0.0019	−2.30	10,870.2	0.0047	1.41			
10,115.3	0.0019	2.88	4479.9	0.0052	1.78			
14,092.6	0.0027	−2.11	6127.2	0.0064	1.60			
3470.1	0.0033	−1.97	7407.8	0.0089	−2.55			
4693.8	0.0034	1.82	1231.8	0.0115	−1.03			
7407.8	0.0044	2.65	11,416.6	0.0156	−1.99			
11,416.6	0.0065	2.87	10,115.3	0.0243	−1.80			
9443.9	0.0094	−1.84	11,073.6	0.0848	−1.32			
10,871.5	0.0108	−2.03	8632.6	0.0883	1.44			
7998.9	0.0186	−1.59						
1470.8	0.0466	−1.35						
11,812.4	0.0655	−1.97						

*m*/*z*—mass value; *p*—peak comparison of the respective groups; FC—fold change; “+” denotes fold change (upregulated); “−“denotes fold change (downregulated).

**Table 3:** Potential protein markers identified for mozzarella cheese in this study.

Predicted *m*/*z*	Theoretical *m*/*z*	Farmstead	Company	Import	Suggested Peptide	References
1101.5	1102.5	+	+	-	as1-CNf(165–173)	[28]
					β-CNf (178–186)	[36]
1231.8	1231.6	+	+	-	β-CNf (123–133)	[36]
	1232.6				as2-CNf(88–96	
3976.2	3975.1	-	+	+	κ-CNf(43–76)	[36]
	3975.8				β-CNf(1–32)	[37,38]
4024.7	4024	+	+	-	ß-CNf(59–96)	[27,39]
	4025				ß-CNf(57–93)	[40]
4233.6	4235	-	+	+	as1-CNf(1–36)	[27,28]
	4237				as1-CNf(80–114)	[40]
7407.8 *	-	+	-	-	-	-
11,416.6 *	-	+	-	-	-	-

* Denotes protein markers identified for authentication of Korean farmstead cheeses.

## Data Availability

All data are provided in the manuscript.
